# To dig oneself into a hole or to dig the tunnel to the core? A study on the Swartz sheath support strategy for patent foramen ovale closure in migraine patients

**DOI:** 10.1515/jtim-2026-0024

**Published:** 2026-06-13

**Authors:** Jianing Fan, Jiaxin Miao, Qixin Chen, Zhenzhen Li, Lei Zhang, Mingfei Li, Dandan Chen, Wenzhi Pan, Xiaochun Zhang, Daxin Zhou, Junbo Ge

**Affiliations:** Department of Cardiology, Zhongshan Hospital, Fudan University; National Clinical Research Center for Interventional Medicine, Shanghai, China

**Keywords:** patent foramen ovale, migraine, swartz sheath, intervention strategy

## Abstract

**Objectives:**

Patent foramen ovale (PFO) closure effectively alleviates symptoms in patients with migraines; however, multipurpose angiographic catheters often encounter difficulty in passing through a small shunt PFO. Here, we present a procedural approach for the treatment of small-shunt PFO and mid-term therapeutic outcomes.

**Methods:**

Patients with migraine and concomitant PFO who visited our hospital between January 2020 and December 2022 were enrolled and grouped according to the application of the Swartz sheath support strategy (SSSS). Baseline characteristics, examination results, and surgical information were recorded. All patients were followed up for > 12 months, with the primary clinical endpoints being relief and cessation of migraine.

**Results:**

A total of 285 patients were included, of whom 82 (28.8%) underwent the SSSS. No surgery-related complications were observed in the SSSS group. The relief (*P* = 0.823) and cessation rates of headaches (*P* = 0.742) were similar between the SSSS and non-SSSS groups. However, the absolute reduction in migraine burden was greater in the SSSS group (*P* = 0.002). After propensity score overlap weighting, no significant differences in migraine outcomes were observed between the two groups. A stronger intensity of attacks (odds ratio [OR]: 4.52, 95% confidence interval [CI]: 1.33–15.30, *P* = 0.015) and medium-to-large degree residual shunt (OR: 4.52, 95% CI: 1.38–57.41, *P* = 0.020) were associated with migraine relief.

**Conclusions:**

The SSSS is a safe and feasible technique for PFO closure in patients with migraine and difficult catheter passage, with postoperative improvement in migraine symptoms observed.

## Introduction

Migraine is a leading cause of disability worldwide and imposes a significant burden on both healthcare systems and society. The etiology of migraine remains complex, and effective treatments are still lacking.^[1,2]^ Recent studies have suggested that patent foramen ovale (PFO) may play a role in migraine pathophysiology, though the mechanisms remain unclear. PFO closure significantly reduces migraine frequency, with relief rates reaching 50%–80%.^[3,4]^

The key step in PFO closure is the successful and safe passage of the catheter through the foramen ovale to the left atrium, followed by deployment and release of the occluder at the appropriate position. A transseptal puncture technique has also been proposed for PFO treatment when catheter passage is difficult. However, this strategy increases the extent of cardiac injury and is associated with a higher risk of residual shunt and ischemic events.^[5]^

The outer Swartz sheath provides additional support for the catheter, aiding in the separation of the primary and secondary septa. This compromised approach increases the likelihood of successful guidewire passage and reduces the risk of atrial septal dissection or damage. In this study, we introduce the Swartz sheath support technique for PFO closure, detailing the operational process and mid-term treatment outcomes.

## Materials and methods

### Study population and classification

This retrospective study included patients with migraine aged 18–55 years who underwent PFO closure at Zhongshan Hospital between January 2020 and December 2022. The exclusion criteria were as follows: (1) inability to pass the catheter through the PFO, (2) incomplete clinical information, and (3) lack of postoperative follow-up. Patients were categorized into the Swartz Sheath Support Strategy (SSSS) and non-SSSS groups based on whether the SSSS was applied. This study was approved by the Ethics Committee of Zhongshan Hospital, Fudan University. Informed consent was obtained from all participants.

### Diagnostic assessment

A multidisciplinary team assessed all patients. Patients with migraine without apparent neurological abnormalities (*e.g*., focal deficits, seizure disorders, or imaging-confirmed lesions such as ischemic stroke, intracranial hemorrhage, or space-occupying lesions with clear etiology) were identified through neurological evaluation, complemented by neuroimaging (computed tomography [CT] or magnetic resonance imaging [MRI] when clinically indicated), and referred to the cardiology department. Patients with a right-to-left shunt (RLS) identified *via* transthoracic echocardiography (TTE) underwent transesophageal echocardiography (TEE) to exclude false-positive findings and further evaluate the PFO anatomy. The degree of shunting was classified both at rest and during the Valsalva maneuver as small (1–10 microbubbles [MB]), medium (10–30 MB), or large ( > 30 MB). TEE was performed to detect abnormal intracardiac shunts and assess the PFO morphology based on clear visualization of the tunnel and its opening, along with measurements of the tunnel length and height. This technology is used to exclude pulmonary arteriovenous malformations using pulmonary CT angiography to assess pulmonary shunting.

### Surgical procedure

The SSSS was integrated into the standard surgical procedure to enhance the probability of successful guidewire passage. The steps were as follows:

1. The patient was placed in the supine position on the operating table. After local conscious sedation and femoral vein puncture, a Swartz sheath was advanced to the top of the right atrium.
2. Fluoroscopy in the left anterior oblique angle was performed to distinguish the atria. The inner core was exchanged with a multi-purpose angiographic (MPA) catheter (Supplementary Figure 1). A contrast agent was injected into the MPA catheter and gradually pulled toward the atrial floor to the opening of the PFO. The opening of the PFO was observed using digital subtraction angiography (DSA).
3. The operator fine-tuned the catheter direction while advancing the super-slick guidewire, instructed the patient to cooperate by coughing, and performed the Valsalva maneuver to increase the guidewire passage rate.
4. Once the guidewire was passed through, the site was confirmed using TTE or intracardiac echocardiography (ICE).
5. A delivery track for the occluder was established, and the occluder was gradually deployed.
6. The shape, position, and pericardial effusion status of the occluder were observed using DSA and TTE. The intracardiac bubble test was performed to check for residual shunting. The occluder was removed after confirming the absence of significant abnormalities. The patient received dual antiplatelet therapy (aspirin 100 mg once daily and clopidogrel 75 mg once daily) for 1 month after the procedure, followed by single antiplatelet therapy (aspirin 100 mg once daily) for 5 months.

**Figure 1:**
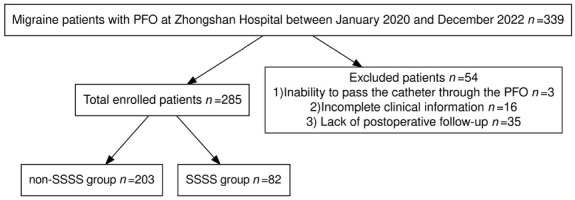
Patient enrollment flowchart. PFO: patent foramen ovale; SSSS: Swartz sheath support strategy.

### Follow-up

Follow-up visits and telephone consultations were conducted 12 months postoperatively to monitor migraine symptoms. Adverse events occurring during and after the procedure, along with postoperative TTE and electrocardiogram (ECG) assessments, were evaluated at 1 day and 1, 6, and 12 months after device implantation; a residual right-to-left shunt was defined as a positive bubble study on TTE at 6 months after PFO closure.^[6]^

### Therapeutic evaluation

The treatment efficacy was assessed based on the following criteria outlined by Eyal *et al*.^[6]^: (1) Frequency of migraine attacks (number of monthly migraine episodes); (2) duration of migraine (average length of headache episodes); (3) migraine burden (product of the attack frequency and average attack duration); (4) alleviation (a reduction in migraine burden by > 50%); and (5) migraine termination. The primary endpoints were migraine alleviation and determinant.

### Statistical analysis

Continuous variables following a normal distribution are expressed as the mean ± standard deviation (SD), while categorical variables are presented as proportions (%). Comparisons between the two groups were made using Student’s *t*-test, paired Student’s *t*-test, and *χ*^2^ test. The Fisher’s exact probability test was used to compare groups if the frequency was < 5. Single-and multivariate logistic regression analyses were performed to screen candidates and identify the independent predictors of migraine symptom improvement. Two-tailed tests were performed throughout the study. We applied propensity score overlap weighting to compare the treatment effects between the two surgical strategies. First, a logistic regression model was fitted to estimate each patient’s propensity for receiving SSSS versus non-SSSS. Individual overlap weights were then calculated by assigning SSSS patients a weight equal to the predicted probability of not receiving SSSS, and non-SSSS patients a weight equal to the predicted probability of receiving SSSS. Finally, overlap-weighted linear regression models (for continuous outcomes) or overlap-weighted logistic regression models (for binary outcomes) were used to estimate the treatment effect between the two groups.

## Results

### Baseline characteristics of the SSSS and non-SSSS groups

A total of 285 patients were included, comprising 203 (71.2%) in the non-SSSS group and 82 (28.8%) in the SSSS group (Figure 1). No significant differences in demographic characteristics were observed between the two groups. Regarding headache features, the migraine burden (59.5 ± 61.7 *vs*. 89.1 ± 86.7 h/mo, *P* = 0.039) and intensity of attacks (7.9 ± 1.3 *vs*. 6.8 ± 1.1 h/mo, *P* = 0.046) were significantly higher in the SSSS group. Additionally, the proportion of chronic migraine was significantly higher in the SSSS group (13.9% *vs*. 40.0%, *P* < 0.001). The incidence of vomiting and reliance on medication for relief was also significantly higher in the SSSS group (52.2% *vs*. 71.9%, *P* = 0.016) (Table 1).

**Table 1:** Baseline clinical characteristics of the patients

Characteristics	Non-SSSS Group (*n* = 203)	SSSS Group (*n* = 82)	*P*-value
General characteristics			
Age (years)	37.3 ± 10.8	36.3 ± 10.1	0.543
Female	154 (75.8%)	65 (79.2%)	0.865
Hypertension	10 (4.9%)	6 (7.3%)	0.461
Diabetes	4 (1.9%)	2 (2.4%)	0.932
Hyperlipidemia	15 (7.3%)	6 (7.3%)	0.985
Family history of migraine	52 (25.6%)	22 (26.8%)	0.885
History of CVA/TIA	56 (27.6%)	16 (19.5%)	0.313
Headache characteristics			
Age at First Headache (years)	23.2 ± 11.7	23.5 ± 8.2	0.844
Disease duration (years)	13.1 ± 10.6	12.5 ± 10.4	0.855
Headache frequency (times/mo)	6.3 ± 7.4	8.6 ± 8.1	0.164
Headache duration (h)	12.2 ± 9.6	13.4 ± 9.7	0.415
Migraine burden (h/mo)	59.5 ± 61.7	89.1 ± 86.7	0.039
Intensity of attacks (VAS score)	6.8 ± 1.1	7.9 ±1.3	0.046
Chronic migraine	28 (13.8%)	32 (39.0%)	<0.001
Migraine with aura	36 (17.7%)	10 (12.9%)	0.303
Vomiting	115 (56.7%)	57 (69.5%)	0.109
Reliance on medication for relief	106 (52.2%)	59 (71.9%)	0.016
Migraine Triggers			
Emotional Stress	63 (31.0%)	27 (32.9%)	0.864
Intense physical activity	52 (25.6%)	13 (15.8%)	0.155
Sleep disturbances	126 (62.1%)	51 (62.2%)	0.974
Fatigue	157 (77.3%)	60 (73.1%)	0.525
Temperature changes	47 (23.2%)	27 (32.9%)	0.226

CVA: cerebrovascular accident; TIA: transient ischemic attack; VAS: visual analog scale; SSSS: Swartz sheath support strategy.

### Preoperative and intraoperative examination

In the TEE examination, the tunnel width in the SSSS group was significantly smaller (1.2 ± 0.9 *vs*. 0.7 ± 0.6 mm, *P* < 0.001). However, there were no significant differences in tunnel length between the SSSS and non-SSSS groups (9.9 ± 4.4 *vs*. 9.2 ± 3.6 mm, *P* = 0.424). In the TTE examination, significant differences were observed in the RLS grading under the Valsalva maneuver (*P* < 0.001). Specifically, the proportion of patients with grade I (50.0%) and grade II (42.7%) SSSS was significantly higher, whereas most patients in the non-SSSS group (72.9%) were classified as having grade III. Additionally, the proportion of permanent RLS was significantly lower in the SSSS group (40.3% *vs*. 57.1%, *P* < 0.001). Regarding other echocardiographic parameters, the left atrial diameter was significantly smaller in the SSSS group (32.0 ± 3.6 *vs*. 33.3 ± 4.0 mm, *P* = 0.045), but there were no significant differences in left ventricular end-diastolic diameter, interventricular septum thickness, left ventricular ejection fraction, or pulmonary artery systolic pressure. In the right heart catheterization results, the pulmonary artery systolic pressure was slightly higher in the SSSS group (26.3 ± 6.9 *vs*. 23.4 ± 5.0 mmHg, *P* = 0.083), and the right ventricular mean pressure was lower (4.6 ± 2.7 *vs*. 6.0 ± 3.8 mmHg, *P* = 0.07). The left-right atrial systolic pressure difference was higher (7.1 ± 4.0 *vs*. 6.0 ± 3.7 mmHg, *P* = 0.094), but these differences were not statistically significant. The procedural time in the SSSS group was significantly longer than that in the non-SSSS group (51.8 ± 22.6 *vs*. 38.1 ± 12.9 min, *P* = 0.032) (Table 2).

**Table 2:** Preoperative and intraoperative examination results of the two groups

Characteristic	Non-SSSS Group (*n* = 203)	SSSS Group (*n* = 82)	*P*-value
TEE			
Tunnel length (mm)	9.9 ± 4.4	9.2 ± 3.6	0.424
Tunnel width (mm)	1.2 ± 0.9	0.7 ± 0.6	< 0.001
TTE			
RLS grading under valsalva maneuver			< 0.001
I	22 (18.8%)	41 (50.0%)	
II	33 (16.3%)	35 (42.7%)	
III	148 (72.9%)	6 (7.3%)	
Permanent RLS	116 (57.1%)	33 (40.3%)	< 0.001
Left atrial diameter (mm)	33.3 ± 4.0	32.0 ± 3.6	0.045
Left ventricular end-diastolic diameter (mm)	44.5 ± 3.9	44.7 ± 4.1	0.811
Interventricular septum thickness (mm)	8.1 ± 1.2	8.2 ± 1.0	0.736
Pulmonary artery systolic pressure (mm)	29.6 ± 3.9	30.6 ± 4.5	0.324
Left ventricular ejection fraction (%)	66.6 ± 3.8	66.5 ± 4.5	0.873
Right heart catheterization			
Pulmonary artery systolic pressure (mmHg)	23.4 ± 5.0	26.3 ± 6.9	0.083
Pulmonary artery mean pressure (mmHg)	12.8 ± 3.6	13.3 ± 4.4	0.561
Right ventricular systolic pressure (mmHg)	27 ± 7	28 ± 7	0.636
Right ventricular mean pressure (mmHg)	6.0 ± 3.8	4.6 ± 2.7	0.070
Right atrial systolic pressure (mmHg)	6.5 ± 3.0	6.0 ± 3.0	0.483
Right atrial mean pressure (mmHg)	2.8 ± 3.6	2.5 ± 2.7	0.636
Left atrial systolic pressure (mmHg)	12.4 ± 4.9	12.9 ± 4.0	0.653
Left atrial mean pressure (mmHg)	5.6 ± 2.5	5.9 ± 3.0	0.546
Left-right atrial systolic pressure difference (mmHg)	6.0 ± 3.7	7.1 ± 4.0	0.094
Left-right atrial mean pressure difference (mmHg)	2.9 ± 3.1	3.5 ± 1.9	0.148
Procedure duration (min)	38.1 ± 12.9	51.8 ± 22.6	0.032

TEE: transesophageal echocardiography; TTE: transthoracic echocardiography; RLS: right-to-left shunt; SSSS: Swartz sheath support strategy.

### Intraoperative imaging of the SSSS

The key steps in using the SSSS for PFO closure are illustrated in Videos 1–3 (as Supplementary Materials). The operator confirmed the opening of the PFO using DSA, TTE, or ICE. A Swartz sheath was then used to advance the guidewire. After the occluder was deployed, an intracardiac bubble test was performed to confirm correct guidewire passage through the PFO, as well as the morphology and location of the PFO.

### Postoperative relief outcomes in the SSSS and non-SSSS groups

**Figure 2:**
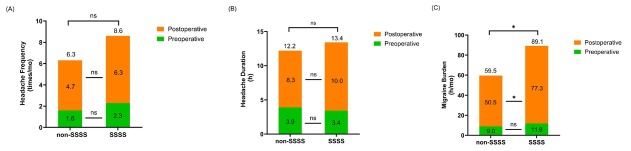
Improvement in migraine symptoms before and after PFO closure in both groups. (A) Changes in headache frequency before and after surgery in both groups. (B) Changes in headache duration before and after surgery in both groups. (C) Changes in migraine burden before and after surgery in both groups. ns: not significant; ^*^P < 0.001. PFO: patent foramen ovale.

**Figure 3:**
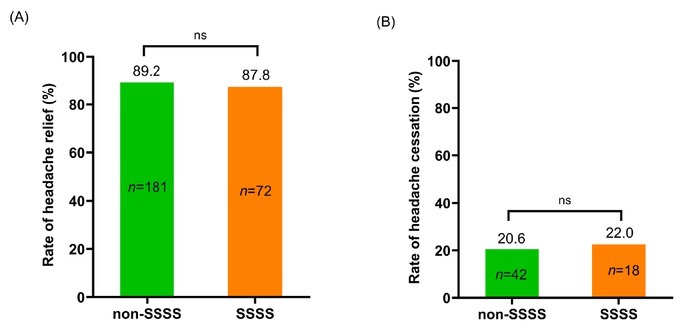
Comparison of treatment outcomes between the two groups. (A) Migraine relief rate in both groups. (B) Migraine termination rate in both groups. ns: not significant. SSSS: Swartz sheath support strategy.

In the unweighted analysis, the reduction in migraine burden was significantly greater in the SSSS group than in the non-SSSS group (77.3 ± 87.1 *vs*. 50.5 ± 58.7 h/mo, *P* = 0.002). There was also a small difference in the reduction of postoperative headache frequency (4.7 ± 4.6 *vs*. 6.3 ± 5.6 times/mo, *P* = 0.074), although this did not reach statistical significance. Headache relief and headache cessation rates were comparable between the two groups (relief: 87.8% *vs*. 89.2%, *P* = 0.823; cessation: 22.0% *vs*. 20.6%, *P* = 0.742; Table 3; Figures 2 and 3).

**Table 3:** Headache improvement at 12-month follow-up in the two groups of patients

Characteristics	Non-SSSS Group (*n* = 203)	SSSS Group (*n* = 82)	*P*-value
Postoperative headache duration (h)	3.9 ± 4.9	3.4 ± 5.1	0.638
Postoperative headache frequency (times/mo)	1.6 ± 3.3	1.3 ± 2.1	0.268
Postoperative headache burden (h/mo)	9.0 ± 14.3	11.8 ± 22.7	0.504
Reduction in headache duration (h)	8.3 ± 12.5	10 ± 9.7	0.463
Reduction in postoperative headache frequency (times/mo)	4.7 ± 5.6	6.3 ± 4.6	0.074
Reduction in migraine burden (h/mo)	50.5 ± 58.7	77.3 ± 87.1	0.002
Headache relief	181 (89.2%)	72 (87.8%)	0.823
Headache cessation	42 (20.6%)	18 (22.0%)	0.742

SSSS: Swartz sheath support strategy.

**Figure 4:**
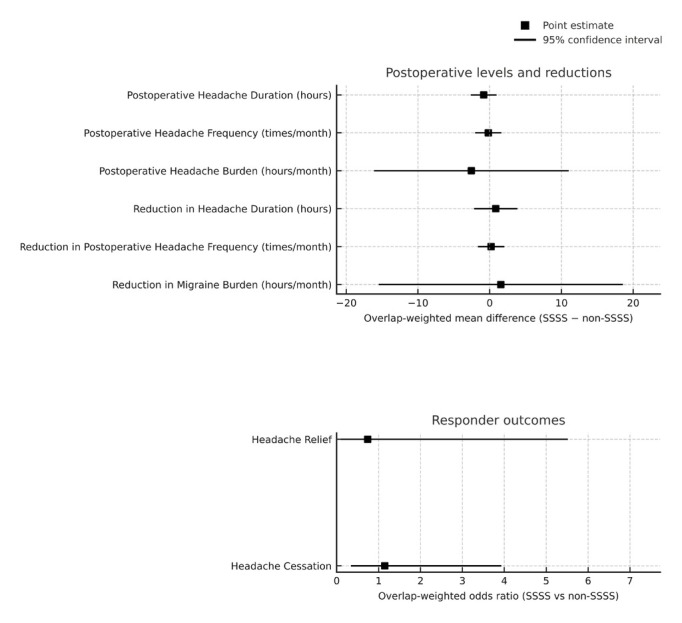
Migraine improvement in the SSSS and non-SSSS groups after propensity score overlap weighting.

In the overlap-weighted analysis, 16 covariates were used to balance the characteristics of the two cohorts, and all covariate means were completely balanced (Supplementary Figure 2). The postoperative headache duration, frequency, and overall burden, as well as the reductions in these measures, were similar between the overlap-weighted SSSS and non-SSSS groups (all *P* > 0.05; Table 4 and Figure 4).

**Table 4:** Overlap-weighted comparison of migraine outcomes between SSSS and non-SSSS groups

Outcome	Non-SSSS (weighted mean ± SD or %)	SSSS (weighted mean ± SD or %)	Effect measure	95% CI	*P*-value
Postoperative Headache Duration (h)	3.55 ± 4.39	2.71 ± 4.65	0.84^*^	-2.65 to 0.97	0.363
Postoperative Headache Frequency (times/mo)	2.35 ± 4.49	2.16 ± 4.67	0.19^*^	-2.03 to 1.64	0.835
Postoperative Headache Burden (h/mo)	11.26 ± 37.62	9.71 ± 23.65	2.55^*^	-16.12 to 11.02	0.239
Reduction in Headache Duration (h)	8.69 ± 8.18	9.53 ± 6.88	0.84^*^	-2.18 to 3.86	0.586
Reduction in Postoperative Headache Frequency (times/mo)	4.61 ± 4.67	4.81 ± 4.56	0.19^*^	-1.65 to 2.04	0.836
Reduction in Migraine Burden (h/mo)	59.50 ± 50.33	61.05 ± 66.22	1.55^*^	-15.48 to 18.57	0.529
Headache Relief	92.2%	89.7%	0.738^#^	0.099 to 5.516	0.767
Headache Cessation	26.7%	29.3%	1.146^#^	0.338 to 3.931	0.360

Continuous outcomes were analysed using overlap-weighted linear regression, and binary outcomes were analysed using overlap-weighted logistic regression. For continuous outcomes, the effect measure is the difference in means^*^; for binary outcomes, the effect measure is the odds ratio^#^. SSSS: Swartz sheath support strategy.

### Predictive factors for migraine improvement

In the univariable analysis, the right ventricular mean pressure (odds ratio [OR]: 1.24, 95% confidence interval [CI]: 1.07–1.42, *P* = 0.003), tunnel width (OR: 2.41, 95% CI: 1.17–6.38, *P* = 0.047), and RLS grading (OR: 11.86, 95% CI: 1.55–90.84, *P* = 0.017) were significantly associated with migraine relief. Specifically, a lower right ventricular mean pressure and lower RLS grade increased the odds of migraine relief. Additionally, the intensity of attacks, measured by the VAS score, was significantly associated with migraine relief (OR: 5.34, 95% CI: 1.70–16.80, *P* = 0.004), indicating that stronger pain intensity was correlated with a higher likelihood of migraine relief. In the multivariable analysis, the intensity of attacks (OR: 4.52, 95% CI: 1.33–15.30, *P* = 0.015) and medium-to-large degree residual shunt (OR: 5.94, 95% CI: 1.38–57.41, *P* = 0.020) remained significantly associated with migraine relief (Table 5).

**Table 5:** Logistic regression analysis of predictors of alleviation in migraine patients

Characteristics	Univariable (OR)	95% CI	*P*-value	Multivariable (OR)	95% CI	*P*-value
Gender	1.00	0.31, 3.18	0.996			
Age	1.02	0.97, 1.07	0.463			
Interventricular Septum Thickness	1.58	1.00, 2.48	0.05	1.30	0.77, 2.20	0.319
Right Ventricular Mean Pressure	1.24	1.07, 1.42	0.003	1.15	0.98, 1.35	0.091
RLS Grading Under Valsalva Maneuver	11.86	1.55, 90.84	0.017	7.58	0.78, 65.89	0.078
Intensity of Attacks (VAS score)	5.34	1.70, 16.80	0.004	4.52	1.33, 15.30	0.015
Tunnel Width	2.41	1.17, 6.38	0.047	2.84	0.96, 8.36	0.059
Medium to Large Degree Residual Shunt	8.71	1.93, 73.36	0.011	5.94	1.38, 57.41	0.020

VAS: visual analog scale; SSSS: Swartz sheath support strategy.

### Perioperative and mid-term surgical complications in the SSSS and non-SSSS groups

All patients underwent the procedure successfully, with no pericardial effusion or atrial septal tears observed in either group. One patient in the non-SSSS group experienced a transient atrioventricular block during surgery, which resolved intraoperatively; no other perioperative complications were noted. During the 6-month follow-up, residual shunts were detected in 66 patients (23.2%): 52/203 (25.6%) in the non-SSSS group and 14/82 (17.1%) in the SSSS group (*P* = 0.162). Moderate shunt occurred in 17 patients (6.0%; 13/203, 6.4% *vs*. 4/82, 4.9%; *P* = 0.272). Severe shunts were rare (2/285, 0.7%) and occurred only in the non-SSSS group (2/203, 1.0% *vs*. 0/82, 0%; *P* = 0.999). At the 12-month follow-up, repeat bubble testing was performed in patients with residual shunt at 6 months, and residual shunts persisted in 32 patients, corresponding to 11.2% (32/285) of the overall cohort: 24/203 (11.8%) in the non-SSSS group and 8/82 (9.8%) in the SSSS group. Moderate residual shunts were observed in 12 patients (4.2%; 9/203, 4.4% *vs*. 3/82, 3.7%), and a severe residual shunt was documented in only 1/285 (0.4%) patient in the non-SSSS group (1/203, 0.5% *vs*. 0/82, 0%). There were no significant differences between the SSSS and non-SSSS groups in the prevalence or severity of residual shunts at 12 months (all *P* > 0.05). All patients survived, with one patient in the non-SSSS group developing new-onset paroxysmal atrial fibrillation. No other new cardiac events or severe complications were observed in any of the patients.

## Discussion

In this study, we found that the SSSS completed the closure of the majority of high-difficulty PFOs. The SSSS was used exclusively for patients in whom the guidewire could not pass through the tunnel during standard procedures. Among the 48 patients who received the SSSS, only 2 (4.2%) had unsuccessful guidewire passage through the PFO. Both patients underwent intraoperative ICE; however, no intracardiac blood flow was observed.

In terms of safety and efficacy, the SSSS is not inferior to the conventional procedures. During both short- and midterm follow-up, no atrial septal tears, pericardial effusion, or other surgery-related complications were observed in patients treated with SSSS. The frequency of postoperative migraine attacks in the SSSS group was similar to that in the non-SSSS group, and both groups showed significant improvement in headache symptoms. Furthermore, by calculating the absolute improvement in migraine burden, we found that the improvement in the SSSS group was more pronounced. We considered that this difference in efficacy was not a result of the choice of technique, but rather, was influenced by the characteristics of the treated population. Therefore, we applied propensity score overlap weighting, a method that generally achieves superior covariate balance and statistical efficiency compared with other propensity score weighting approaches, to balance the baseline patient characteristics;^[7,8]^ in the overlap-weighted cohort, improvements in migraine outcomes were no longer different between the SSSS and non-SSSS groups.

The majority of patients treated with SSSS had a small shunt PFO and presented with a higher migraine burden, which is consistent with previous studies showing higher Migraine Disability Assessment Questionnaire (MIDAS) scores and attack frequency.^[9,10]^ Chronic migraine (CM) is diagnosed in patients who suffer headaches at least 15 days per month, with at least 8 of those days meeting the criteria for migraine.^[11]^ These patients have higher MIDAS scores and a more severe decline in quality of life than those with episodic migraine.^[12]^ RLS is more common in patients with CM, and the RLS grade in patients with CM is generally lower than that in patients with episodic migraine.^[10,13]^ We also observed a higher proportion of patients with CM in the SSSS group.

We further evaluated the predictive factors for migraine improvement. Higher VAS scores and the absence of medium-to large-degree residual shunts were associated with improved relief from headache symptoms. Previous studies have shown that patients with more severe and frequent baseline migraine are more likely to benefit from PFO closure.^[14,15]^ Additionally, a larger PFO tunnel width and shunt volume were associated with long-term residual shunting and contributed to persistent migraine symptoms postoperatively.^[6,16,17,18,19]^ However, in our population, the PFO tunnel width showed limited predictive value for headache improvement, which may be attributed to discrepancies between TEE measurements and the actual size of the PFO under balloon dilation,^[20]^ as well as differences in population characteristics, examiner assessment standards, and patient cooperation during measurements.

Notably, at our center, patients with larger shunts are more likely to be considered at risk for stroke; thus, alleviating migraine symptoms may not be the primary treatment goal, nor has there been a formal assessment of their potential benefit. In contrast, patients with smaller shunts more often present with frequent and severe migraines and may opt for PFO closure as a palliative treatment. This may be one factor contributing to the differences in headache characteristics between the two groups. However, despite the presence of such selection bias and lack of causal validation, the relief rates in both groups were satisfactory. Moreover, the relief rate in the SSSS group was comparable to that observed in another study that focused on patients with a predominantly small-to-medium shunt PFO.^[6]^ Therefore, for patients with migraine with smaller shunts and more severe symptoms, PFO closure remains an important therapeutic option, further emphasizing the clinical value of the SSSS.

Although the procedural time was longer in the SSSS group, this does not reflect the greater complexity of the SSSS technique. The prolonged duration was primarily due to the anatomical characteristics of the PFO, which increased the difficulty of guidewire passage. In all cases, the SSSS method was employed only after the conventional approaches failed. As a rescue strategy, this technique enabled interventional treatment in 28.8% of patients who otherwise could not have undergone the procedure. Unlike the study by Kang *et al*.,^[21]^ our approach applied only the outer Swartz sheath for support, with the inner core replaced by an MPA. The MPA, with its larger distal cross-sectional area and softer texture than the inner core of the Swartz sheath, provides superior contrast for better localization of the PFO opening. Additionally, this modification reduces the risk of atrial septal tears, thereby enhancing the overall safety of the procedure.

## Limitations

This study has certain limitations. First, this retrospective study relied on patient recall to assess migraine characteristics, which are inherently imprecise and subject to recall bias. Future studies should adopt prospective designs with patient diaries or standardized tools, such as the MIDAS or HIT-6, to ensure reliable data collection. Second, the definition of migraine relief was subjective. Although, as in the method by Eyal *et al*.,^[6]^ using migraine burden as a standard for assessing migraine relief is a scientifically valid approach, different endpoint selections might have influenced the conclusions of the study. Third, as mentioned above, selection bias existed in the process of patient enrollment, which necessitates more rigorous randomized controlled trials to explore the characteristics of the population that benefits from PFO closure. Fourth, baseline differences existed between the groups, and future studies should apply multivariable adjustment or propensity score methods to reduce potential bias. Fifth, following a previous study,^[6]^ a comprehensive bubble test follow-up was performed at ≥ 6 months to ensure a more systematic and complete assessment, considering short-term endothelialization, limited postoperative interventions, and resource limitations. We acknowledge that further efforts to enhance follow-up completeness could help minimize potential biases in future studies. Finally, some predictors in our multivariate analysis showed wide confidence intervals, reflecting uncertainty in the effect estimates due to the limited sample size or rare events. This limitation should be considered when interpreting the strength of these associations.

## Conclusions

The SSSS is a safe and feasible option for PFO closure in patients with migraine. Although postoperative improvement in migraine symptoms was observed in this cohort, prospective or randomized controlled studies with standardized migraine assessments are needed to establish a definitive causal link.

## Supplementary Material

Supplementary Material Details

Supplementary Material Details

## Supplementary Information

Videos are for this article are only available as supplementary materials at the official site of the journal (www.intern-med.com).
