# Supplementary material for: To dig oneself into a hole or to dig the tunnel to the core? A study on the Swartz sheath support strategy for patent foramen ovale closure in migraine patients
Source: J Transl Int Med. 2026 Jun 13;14(3):446–55. doi: 10.1515/jtim-2026-0024 (PMC13320534; doi:10.1515/jtim-2026-0024)
Supplement: Supplementary file 1 — Supplementary Material Details [file jtim-2026-0024_sm.pdf]

Supplementary materials

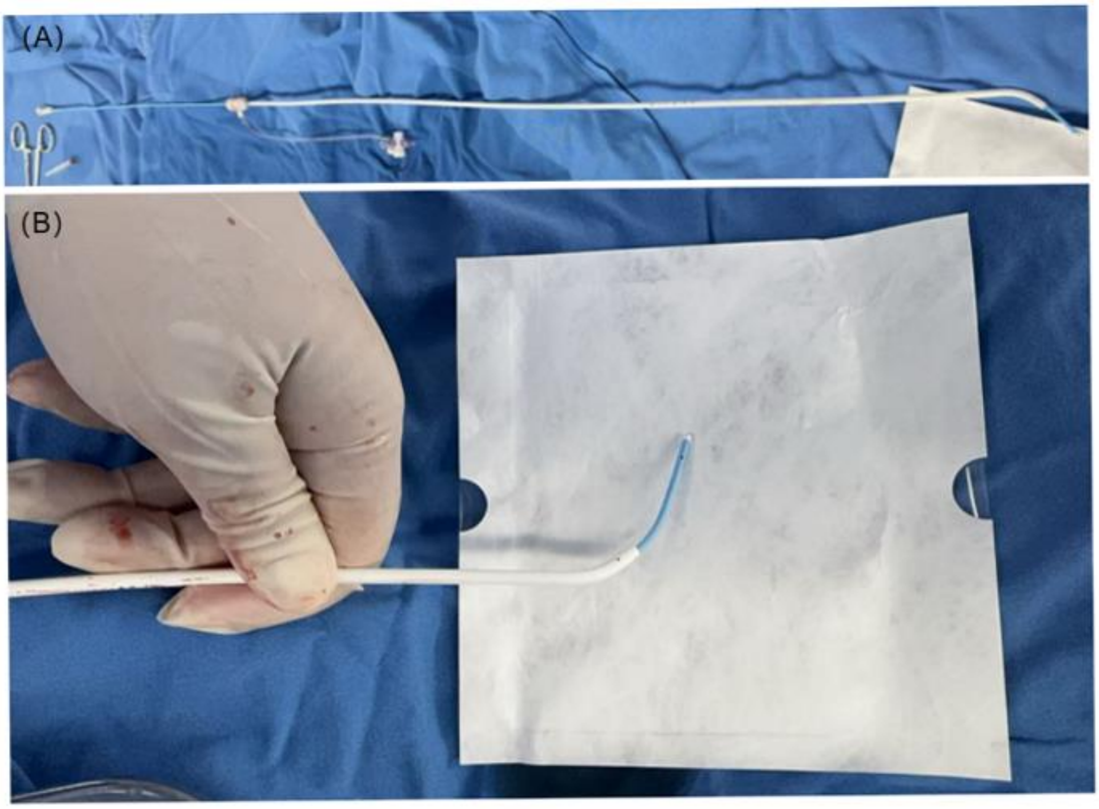

Supplementary Figure S1: Swartz sheath with MPA replaced as the core (A) The entire conveyor system, (B) The head end of the conveyor system.

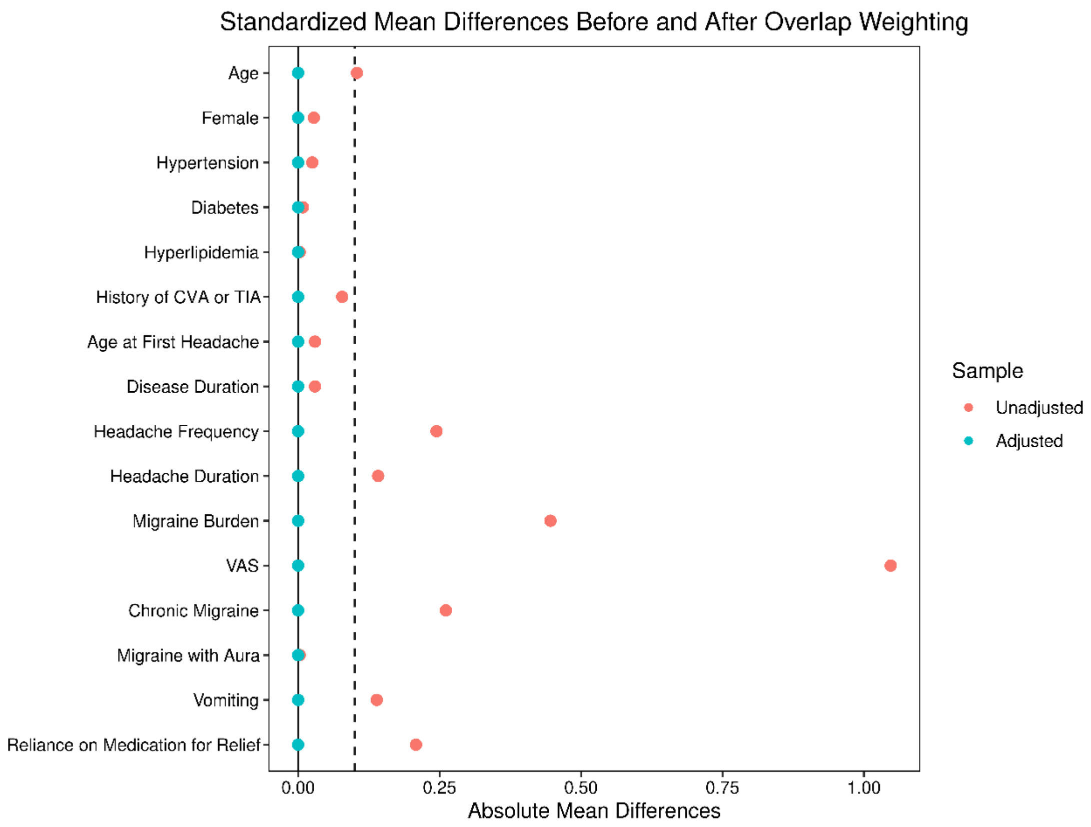

Supplementary Figure S2: Standardized mean differences (SMDs) of covariates between the SSSS and non-SSSS groups before and after overlap weight

Supplementary Table S1: Cohort characteristics, before and after propensity score overlap weighting

	**Unadjusted**				**After overlap weighting**			
	**Non-SSSS Group (n = 203)**	**SSSS Group (n = 82)**	***P***	**SMD**	**Non-SSSS Group (n = 203)**	**SSSS Group (n = 82)**	***P***	**SMD**
Age (years)	37.3 ± 10.8	36.3 10.1	±0.543	0.107	35.52 11.04	±35.52 11.04	±1.000	<0.001
Female (%)	75.8	79.2	0.865	0.068	78.2	78.2	1.000	<0.001
Hypertension (%)	4.9	7.3	0.461	0.103	7.5	7.5	1.000	<0.001
Diabetes (%)	1.9	2.4	0.932	0.058	4.1	4.1	1.000	<0.001
Hyperlipidemia (%)	7.3	7.3	0.985	0.103	7.5	7.5	1.000	<0.001
History of CVA/TIA (%)	27.6	19.5	0.313	0.183	25.5	25.5	1.000	<0.001

	**Unadjusted**				**After overlap weighting**			
	**Non-SSSS Group (*n* = 203)**	**SSSS Group (*n* = 82)**	***P***	**SMD**	**Non-SSSS Group (*n* = 203)**	**SSSS Group (*n* = 82)**	***P***	**SMD**
Age at First Headache (years)	23.2 ± 11.7	23.5 ± 8.2	0.844	0.031	22.73 11.30	±22.73 8.08	±1.000	<0.001
Disease Duration (years)	13.1 ± 10.6	12.5 10.4	±0.855	0.032	12.68 11.04	±12.68 9.78	±1.000	<0.001
Headache Frequency (times/month)	6.3 ± 7.4	8.6 ± 8.1	0.164	0.252	6.96 ± 7.75	6.96 7.48	±1.000	<0.001
Headache Duration (hours)	12.2 ± 9.6	13.4 ± 9.7	0.415	0.142	12.02 8.85	±12.44 9.38	±1.000	<0.001
Migraine Burden (hours/month)	59.5 ± 61.7	89.1 86.7	±0.039	0.408	72.63 81.02	±71.34 70.78	±1.000	<0.001
Intensity of Attacks (VAS score)	6.8 ± 1.1	7.9 ±1.3	0.046	0.906	7.46 ± 1.03	7.46 1.07	±1.000	<0.001
Chronic Migraine (%)	13.8	39.0	< 0.001	0.616	27.3	27.3	1.000	<0.001
Migraine with Aura (%)	17.7	12.9	0.303	0.007	15.7	15.7	1.000	<0.001
Vomiting (%)	56.7	69.5	0.109	0.291	67.1	67.1	1.000	<0.001
Reliance on Medication for Relief (%)	52.2	71.9	0.016	0.440	66.4	66.4	1.000	<0.001

CVA: Cerebrovascular accident, TIA: Transient ischemic attack, VAS: Visual Analog Scale, SSSS: Swartz sheath support strategy, SMD, standardized mean difference.

Video 1: PFO opening localization. (A) During contrast injection with the MPA catheter at the potential PFO opening, a tent-like image appears, with a dissection between the primary and secondary septum. (B) ICE showing the PFO tunnel and blood flow signals (Video 2). PFO: patent foramen ovale; ICE: intracardiac echocardiography.

Video 2: Guidewire passage through the PFO under the Swartz sheath support. (A)

Despite the Swartz sheath support, the guidewire struggled to pass through the tunnel, and the catheter popped out of the tunnel opening. (B) After the catheter popped out, re-angiography was performed to confirm the tunnel opening. (C) The patient was instructed to cough to facilitate tunnel opening, allowing the guidewire to pass smoothly. PFO: patent foramen ovale.

Video 3: Bubble test performed after PFO closure. PFO: patent foramen ovale.
